# Grossesse gémellaire associant une grossesse molaire et un fœtus vivant avec évolution vers mole invasive: à propos de deux cas

**DOI:** 10.11604/pamj.2015.22.24.7150

**Published:** 2015-09-10

**Authors:** Ikram Boubess, Adib Filali, Fayssal Benbrahim, Salma Ouassour, Mokha Tazi, Mohammad Hassan Alami

**Affiliations:** 1 Centre National de la Santé Reproductive, CHU Ibn Sina, Rabat, Maroc

**Keywords:** Grossesse gémellaire, mole hydatiforme, mole invasive, twin pregnancy, hydatidiform mole, invasive mole

## Abstract

La grossesse gémellaire associant une môle complète et une grossesse singleton normale possédant son propre trophoblaste sain est une entité rare. La majorité des études montre que le pronostic d'une telle association comprend un risque un peu plus accru d’évolution vers une tumeur trophoblastique gestationnelle. Nous rapportons deux cas de patientes qui ont consulté pour des métrorragies du premier trimestre et dont l’échographie a objectivé une grossesse gémellaire associant une grossesse molaire et une grossesse évolutive singleton et dont l’évolution était marqué par une mole invasive.

## Introduction

La grossesse gémellaire associant une môle complète à un fœtus vivant (GGAMH) est une entité rare survenant dans 1 sur 22 000 à 1 sur 100 000 grossesses [1]. La poursuite de la grossesse est controversée compte tenu des risques de complications maternelles immédiates et à distance. Nous rapportons à ce sujet deux cas cliniques dont l’évolution était la mole invasive.

## Patient et observation

**Figure 1:**
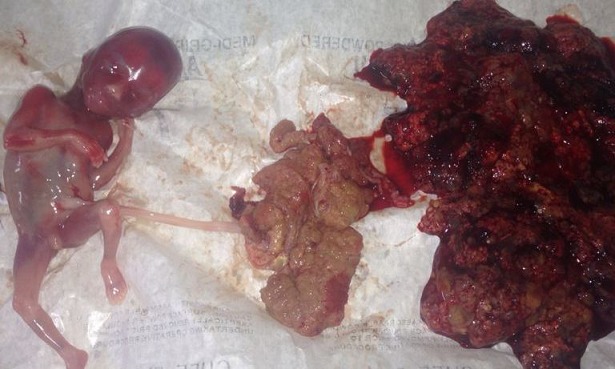
Fœtus vivant de 15 SA avec son propre trophoblaste et une masse vésiculeuse (à droite)

**CAS n°1:** une patiente de 28ans, admise pour métrorragie du deuxième trimestre après une aménorrhée de 15 semaines (SA). L'examen clinique trouve une patiente pâle, un col fermé avec saignement endo-uterin, la hauteur utérine est supérieur à l’âge gestationnel à 18 SA. L’ échographie obstétricale a permis d'objectiver une grossesse gémellaire bichoriale: deux sacs gestationnel dont un avec un fœtus vivant de 15SA, un autre sac gestationnel contenant une cavité amniotique avec un trophoblaste en aspect de nid d'abeille de 13,8cm. Un bilan biologique réalisé en parallèle objectivant un taux de l'hormone gonadotrophine chorionique(beta-hCG)à 112500mUI/L, une anémie hypochrome microcytaire associée à une hyperthyroïdie biologique. La décision d’évacuation utérine par le misoprostol suivi d'une aspiration endo-uterine a été prise sur la base des signes de gravité clinique et du mauvais pronostic de cette association. Le produit d'expulsion était fait d'un fœtus vivant de 15 SA avec son propre trophoblaste associé à une masse vésiculeuse de 13cm (Figure 1) qui à l'examen histologique correspondait à une môle hydatiforme complète. La surveillance de la courbe de beta HCG plasmatique a objectivé une reascension de ce taux six semaine après l'expulsion avec la découverte d'une invasion myomètriale à l’échographie de contrôle.

**Figure 2:**
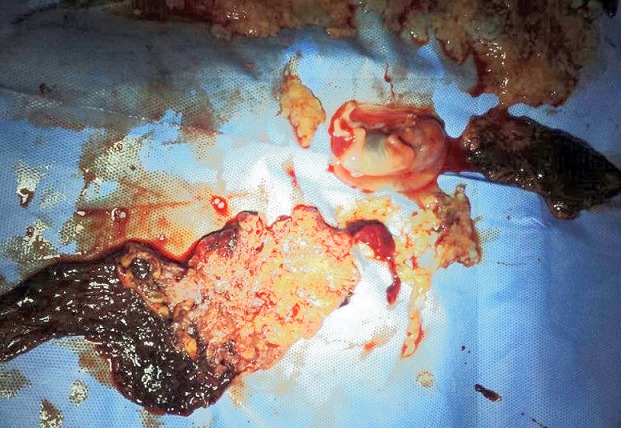
Fœtus de 11SA (à droite) avec son propre trophoblaste (en bas) et des multiples vésicules molaires (en haut)

**Figure 3:**
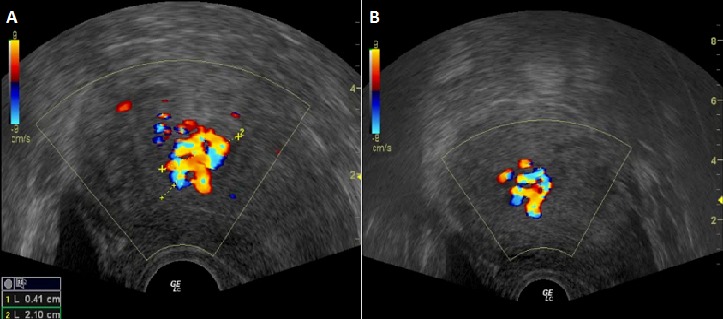
Échographie pelvienne avec doppler couleur objectivant une invasion du myométre sur le mur antérieur de l'utérus

**CAS n°2:** une primigeste de 30 ans, admise pour des métrorragies du premier trimestre. L'examen clinique a objectivé un utérus de 14 SA avec un saignement minime endo-uterin. L’échographie obstétricale a objectivé une grossesse gémellaire bichoriale avec un sac gestationnel contenant un fœtus vivant de 11 SA et un autre sac gestationnel contenant une cavité amniotique avec une masse en nid d'abeille de 5,8x5,6cm de diamétre. Le bilan biologique a objectivé un taux de béta-hCG à 160850 mUI/L, une anémie hypochrome microcytaire avec un bilan thyroïdien normal. Une interruption médicale de la grossesse par le misoprostol et l'aspiration endo-utérine était décidée avec expulsion en premier temps d'un fœtus de 11SA avec son trophoblaste suivi d'expulsion de multiple vésicules de différents taille (Figure 2). L'examen histologique de la masse vésiculeuse était en faveur d'une mole hydatiforme complète. La surveillance du taux BHCG a montré une stagnation de ses valeurs sur 3 semaines avec des signes d'invasion myométriale à l’échographie doppler (Figure 3).

## Discussion

La môle gémellaire avec coexistence d'un œuf avec fœtus vivant et d'une grossesse môlaire est un événement relativement inhabituel. La prise en charge de ces grossesses est difficile, de part les complications telles que la mort fœtale, les saignements, la prééclampsie, l'hyperthyroïdie, et le risque d’évolution vers une tumeur trophoblastique gestationelle [2, 3]. Ce risque de tumeur gestationelle trophoblastique est le plus redouté. L'incidence de tumeur trophoblastique gestationelle semble plus importante dans le cas d'association d'une môle hydatiforme complète avec une grossesse singleton normale selon la majorité des études publiées [2, 4, 5] avec des taux variant entre 50 à 57%. Par contre sebire et al [6] qui a publié la plus grande série a trouvé un taux similaire entre les moles hydatiformes simples 16% et les moles hydatiformes associées à une grossesse gémellaire 19%. Des échographies répétées permettent de suivre l’évolution du placenta molaire et d’évoquer une invasion myométriale au doppler couleur [7]. Les recommandations de prise en charge de GGAMH ne sont pas encore codifiées mais certains auteurs suggèrent que la grossesse peut être menée à terme si le diagnostic est fait tardivement et en absence de complications [8] et que la probabilité d'obtenir une naissance vivante variant entre 16 et 56% [9]. Pour les cas ou le diagnostic de GGAMH était fait à un âge précoce, l'interruption de la grossesse est souvent de mise [10]. Dans le cas de nos deux patientes, nous avons opté pour l'arrêt de la grossesse vu le très jeune âge de la grossesse chez les deux patientes en plus du syndrome anémique et l'hyperthoidie chez la première ainsi que les complications qui auraient pu survenir si on avaient accepté de conserver la grossesse. L’évolution de ces deux moles était vers une tumeur trophoblastique gestationelle classé à bas risque selon la classification de la fédération internationale de gynécologie obstétrique 2000 [10] et une monochimiothérapie à base de méthotrexate a été préconisée avec une bonne évolution clinique et biologique.

## Conclusion

Le risque de complications et notamment d'invasion myometriale est augmenté en cas de grossesse gémellaire associant une mole hydatiforme à une grossesse évolutive en comparaison à la môle hydatiforme complète isolée. On ne dispose pas aujourd'hui d'arguments suffisants pour recommander une interruption de ces grossesses ou d'accepter une surveillance malgré les risques maternelles, pour nos deux malades nous avons adopté la première attitude qui semble plus sécurisante.
